# The genome sequence of a pallopterid fly, *Toxonevra muliebris *(Harris, 1780)

**DOI:** 10.12688/wellcomeopenres.23670.1

**Published:** 2025-02-10

**Authors:** Maxwell V. L. Barclay, Gavin R. Broad, Olga Sivell, Natural History Museum Genome Acquisition Lab, Darwin Tree of Life Barcoding collective, Wellcome Sanger Institute Tree of Life Management, Samples and Laboratory team, Wellcome Sanger Institute Scientific Operations: Sequencing Operations, Wellcome Sanger Institute Tree of Life Core Informatics team, Tree of Life Core Informatics collective, Darwin Tree of Life Consortium

**Affiliations:** 1 Natural History Museum, London, England, UK

**Keywords:** Toxonevra muliebris, pallopterid fly, genome sequence, chromosomal, Diptera

## Abstract

We present a genome assembly from an individual male specimen of *Toxonevra muliebris* (pallopterid fly; Arthropoda; Insecta; Diptera; Pallopteridae). The genome sequence has a total length of 491.40 megabases. Most of the assembly (99.07%) is scaffolded into 6 chromosomal pseudomolecules, including the X and Y sex chromosomes. The mitochondrial genome has also been assembled and is 16.18 kilobases in length. Gene annotation of this assembly on Ensembl identified 21,433 protein-coding genes.

## Species taxonomy

Eukaryota; Opisthokonta; Metazoa; Eumetazoa; Bilateria; Protostomia; Ecdysozoa; Panarthropoda; Arthropoda; Mandibulata; Pancrustacea; Hexapoda; Insecta; Dicondylia; Pterygota; Neoptera; Endopterygota; Diptera; Brachycera; Muscomorpha; Eremoneura; Cyclorrhapha; Schizophora; Acalyptratae; Tephritoidea; Pallopteridae; *Toxonevra*; *Toxonevra muliebris* (Harris, 1780) (NCBI:txid2725530)

## Background

*Toxonevra muliebris* (Harris, 1780) is a small fly species from the family Pallopteridae. The family was split into several genera by McAlpine (1981), however these have not been accepted by British dipterists, and *muliebris* is still placed in the genus *Palloptera* Fallén, 1820 in the British checklist ( Chandler, 2024).

**Figure 1.**
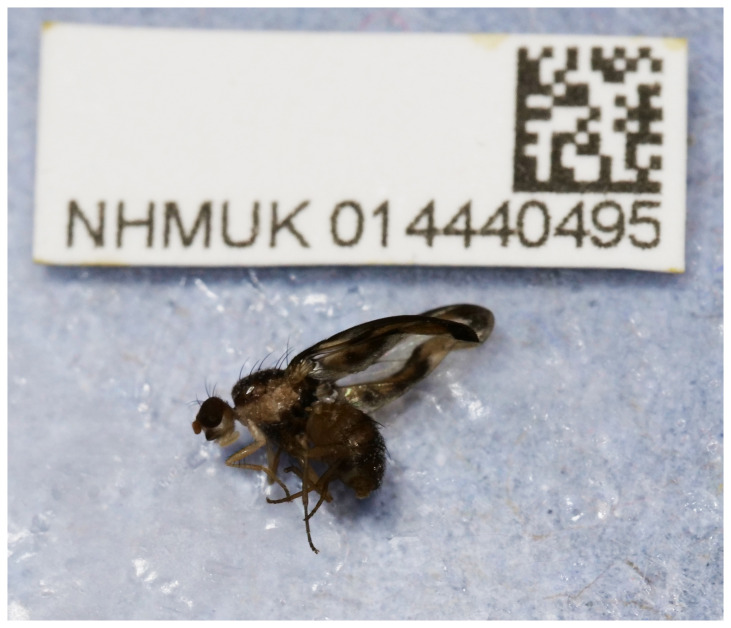
Photograph of the *Toxonevra muliebris* (idToxMuli2) specimen used for genome sequencing.

The species is easy to identify due to the characteristic broad brown band that loops around the wing ( Figure 1). The thorax is brown, with two dark dorsolateral stripes ( Collin, 1951; Stubbs & Clements, 1999). The males extend their wings and vibrate them, which is why the family is sometimes called the flutter flies ( Marshall, 2012; Rotheray, 2014).

*Toxonevra muliebris* is widely distributed in western and northern Europe, from Spain to Germany, Great Britain, Ireland and Denmark (absent from Fennoscandia), with a handful of records from Montenegro, Greece and Turkey. It is also recorded from western Canada (Victoria, BC) ( Cannings & Gibson, 2019; Speight, 1986; Wallace & O’Connor, 1997). In Britain it is widely distributed in the south, becoming less common in northern England and scarce in Scotland ( Weddle, 2019). The adults are active from June to October ( NBN Atlas Partnership, 2024). They are often found indoors, on windows ( Collin, 1951).

Little is known about the biology of *T. muliebris*. The Pallopteridae are likely mostly saprophagous ( Rotheray, 2014). The larvae of *T. muliebris* occur under the bark of conifers and birch, a habit they share with *P. usta*, *P. ustulata* and possibly *P. umbellatarum* ( Chandler, 1991). They have also been recorded from elder ( Parmenter, 1968). The adults also occur indoors, where they may be feeding on the detritus from carpet beetles (Coleoptera, Dermestidae) or the beetle larvae themselves ( Cannings & Gibson, 2019; Jones, 2015; Wallace & O’Connor, 1997).

Molecular phylogeny of the superfamily Tephritoidea was researched by Han and Ro (2016). The *12S*, *16S*, *cytochrome c oxidase I* and *cytochrome c oxidase II* were analysed using Maximum-likelihood and Bayesian inferences. The findings confirm monophyly of the Tephritoidea as well as the monophyly of the family Pallopteridae, excluding *Eurygnathomyia,* which has been placed in the family Eurygnathomyiidae by Griffiths (1972), subsequently synonymised with Pallopteridae and resurrected by Papp (2011), with family status confirmed by the molecular analysis by Han and Ro (2016), although this is not yet accepted by British dipterists ( Chandler, 2024).

The high-quality genome of *T. muliebris* was sequenced as part of the Darwin Tree of Life Project, a collaborative effort to sequence all named eukaryotic species in the Atlantic Archipelago of Britain and Ireland. It will aid research on taxonomy and biology of *T. muliebris* and phylogenetic relationships within Pallopteridae and among related acalyptrate families.

## Genome sequence report

The genome of *Toxonevra muliebris* ( Figure 1) was sequenced using Pacific Biosciences single-molecule HiFi long reads, generating a total of 34.75 Gb (gigabases) from 4.22 million reads, providing an estimated 73-fold coverage. Primary assembly contigs were scaffolded with chromosome conformation Hi-C data, which produced 112.51 Gb from 745.09 million reads. Specimen and sequencing details are summarised in Table 1.

**Table 1.** Specimen and sequencing data for *Toxonevra muliebris*.

Project information			
**Study title**	Toxoneura muliebris (looped flutter fly)		
**Umbrella BioProject**	PRJEB68022		
**Species**	*Toxonevra muliebris*		
**BioSample**	SAMEA112221964		
**NCBI taxonomy ID**	2725530		
Specimen information			
**Technology**	**ToLID**	**BioSample accession**	**Organism part**
**PacBio long read sequencing**	idToxMuli2	SAMEA112222109	abdomen
**Hi-C sequencing**	idToxMuli1	SAMEA7521585	head
Sequencing information			
**Platform**	**Run accession**	**Read count**	**Base count (Gb)**
**Hi-C Illumina NovaSeq 6000**	ERR12245616	7.45e+08	112.51
**PacBio Revio**	ERR12205287	4.22e+06	34.75

Assembly errors, including 23 missing joins or mis-joins and three haplotypic duplications, were corrected by manual curation. This reduced the scaffold number by 1.01%. The final assembly has a total length of 491.40 Mb in 97 sequence scaffolds, with 244 gaps, and a scaffold N50 of 109.6 Mb ( Table 2).

**Table 2.** Genome assembly data for *Toxonevra muliebris*, idToxMuli2.1.

Genome assembly		
Assembly name	idToxMuli2.1	
Assembly accession	GCA_963691655.1	
*Accession of alternate haplotype*	*GCA_963691675.1*	
Span (Mb)	491.40	
Number of contigs	342	
Number of scaffolds	97	
Longest scaffold (Mb)	175.3	
Assembly metrics *		*Benchmark*
Contig N50 length (Mb)	3.5	*≥ 1 Mb*
Scaffold N50 length (Mb)	109.6	*= chromosome N50*
Consensus quality (QV)	61.3	*≥ 40*
*k*-mer completeness	Primary: 84.35%; Alternate: 81.13%; Combined: 99.57%	*≥ 95%*
BUSCO v5.4.3 lineage: diptera_odb10	C:98.7%[S:97.7%,D:1.0%], F:0.3%,M:1.0%,n:3,285	*S > 90%, D < 5%*
Percentage of assembly mapped to chromosomes	99.07%	*≥ 90%*
Sex chromosomes	XY	*localised homologous pairs*
Organelles	Mitochondrial genome: 16.18 kb	*complete single alleles*
Genome annotation of assembly GCA_963691655.1 at Ensembl		
Number of protein-coding genes	21,433	
Number of gene transcripts	22,154	

* Assembly metric benchmarks are adapted from Rhie *et al.* (2021) and the Earth BioGenome Project Report on Assembly Standards September 2024. ** BUSCO scores based on the diptera_odb10 BUSCO set using version 5.4.3. C = complete [S = single copy, D = duplicated], F = fragmented, M = missing, n = number of orthologues in comparison. A full set of BUSCO scores is available at https://blobtoolkit.genomehubs.org/view/Toxonevra_muliebris/dataset/GCA_963691655.1/busco.

**Figure 2.**
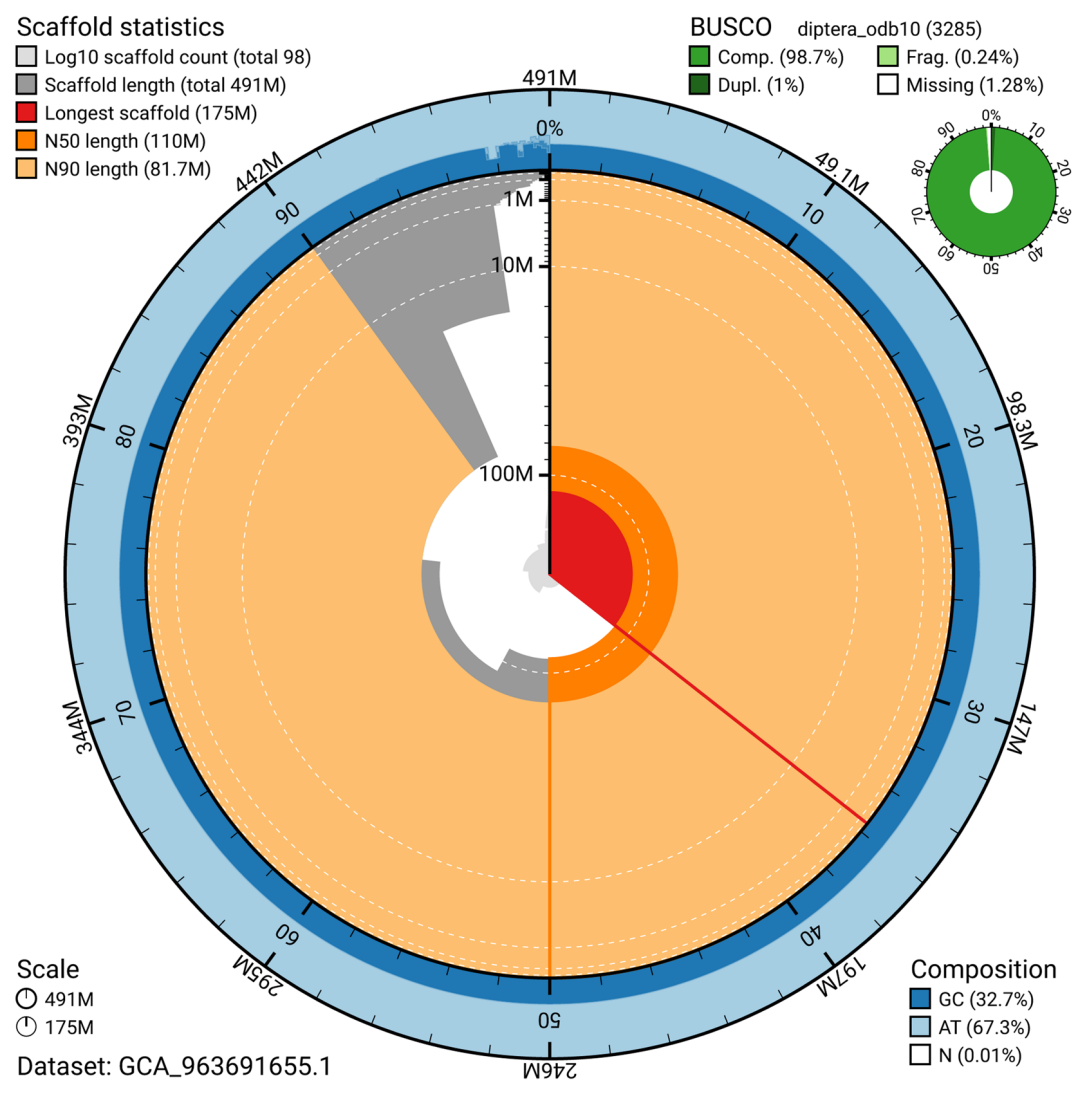
Genome assembly of *Toxonevra muliebris*, idToxMuli2.1: metrics. The BlobToolKit snail plot provides an overview of assembly metrics and BUSCO gene completeness. The circumference represents the length of the whole genome sequence, and the main plot is divided into 1,000 bins around the circumference. The outermost blue tracks display the distribution of GC, AT, and N percentages across the bins. Scaffolds are arranged clockwise from longest to shortest and are depicted in dark grey. The longest scaffold is indicated by the red arc, and the deeper orange and pale orange arcs represent the N50 and N90 lengths. A light grey spiral at the centre shows the cumulative scaffold count on a logarithmic scale. A summary of complete, fragmented, duplicated, and missing BUSCO genes in the diptera_odb10 set is presented at the top right. An interactive version of this figure is available at https://blobtoolkit.genomehubs.org/view/GCA_963691655.1/dataset/GCA_963691655.1/snail.

**Figure 3.**
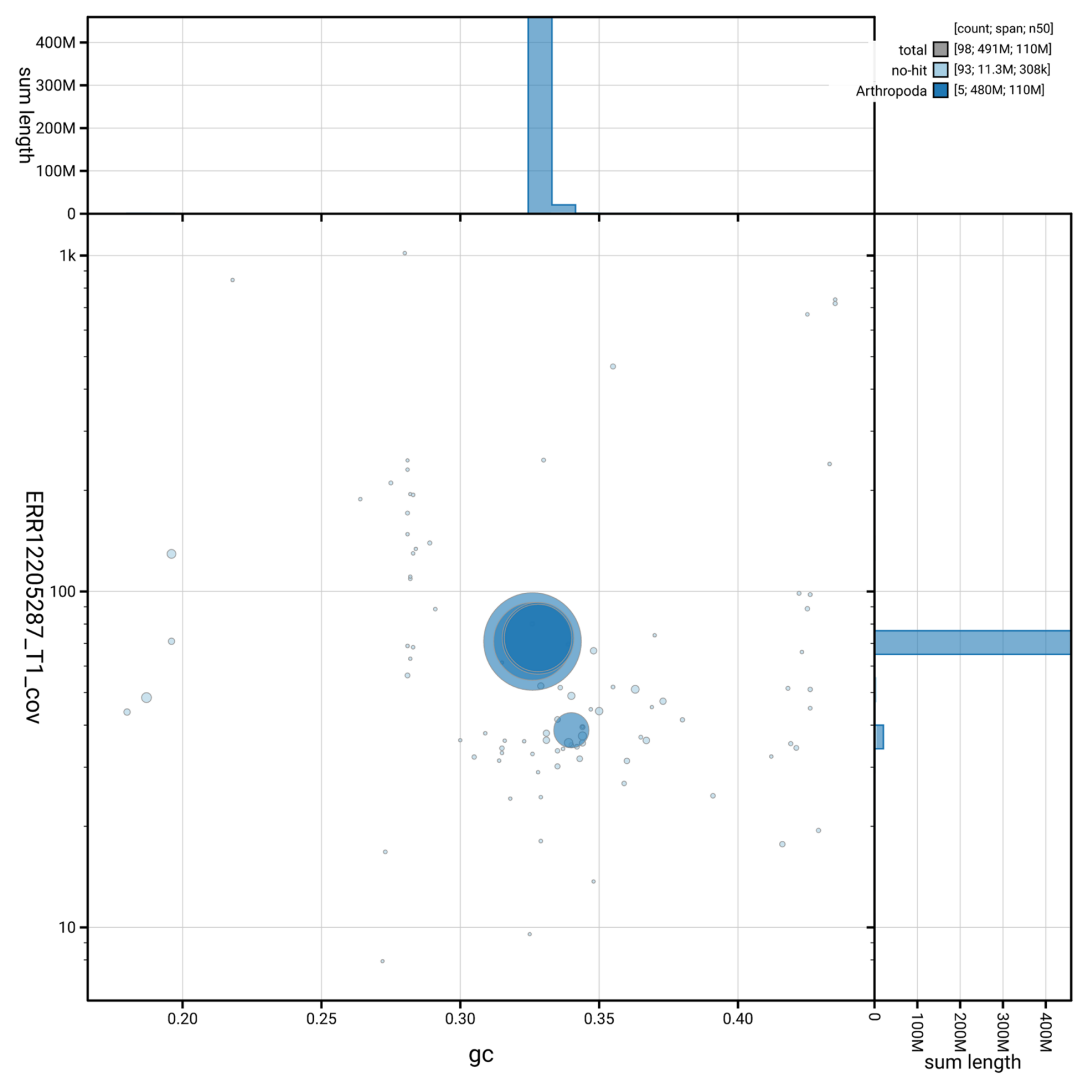
Genome assembly of *Toxonevra muliebris*, idToxMuli2.1: BlobToolKit GC-coverage plot showing sequence coverage (vertical axis) and GC content (horizontal axis). The circles represent scaffolds, with the size proportional to scaffold length and the colour representing phylum membership. The histograms along the axes display the total length of sequences distributed across different levels of coverage and GC content. An interactive version of this figure is available at https://blobtoolkit.genomehubs.org/view/GCA_963691655.1/dataset/GCA_963691655.1/blob.

**Figure 4.**
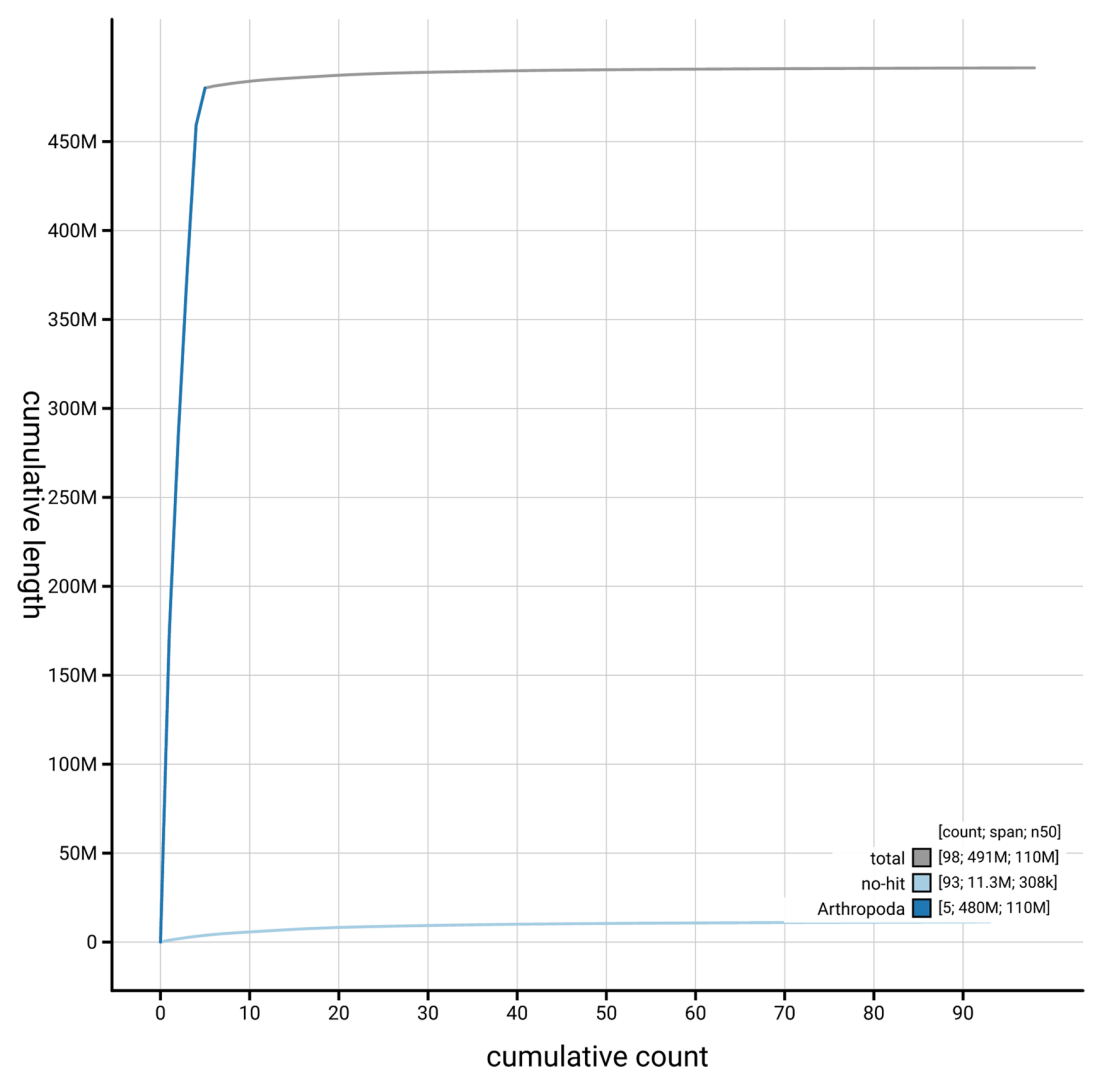
Genome assembly of *Toxonevra muliebris* idToxMuli2.1: BlobToolKit cumulative sequence plot. The grey line shows cumulative length for all scaffolds. Coloured lines show cumulative lengths of scaffolds assigned to each phylum using the buscogenes taxrule. An interactive version of this figure is available at https://blobtoolkit.genomehubs.org/view/GCA_963691655.1/dataset/GCA_963691655.1/cumulative.

The snail plot in Figure 2 provides a summary of the assembly statistics, indicating the distribution of scaffold lengths and other assembly metrics. Figure 3 shows the distribution of scaffolds by GC proportion and coverage. Figure 4 presents a cumulative assembly plot, with separate curves representing different scaffold subsets assigned to various phyla, illustrating the completeness of the assembly.

**Figure 5.**
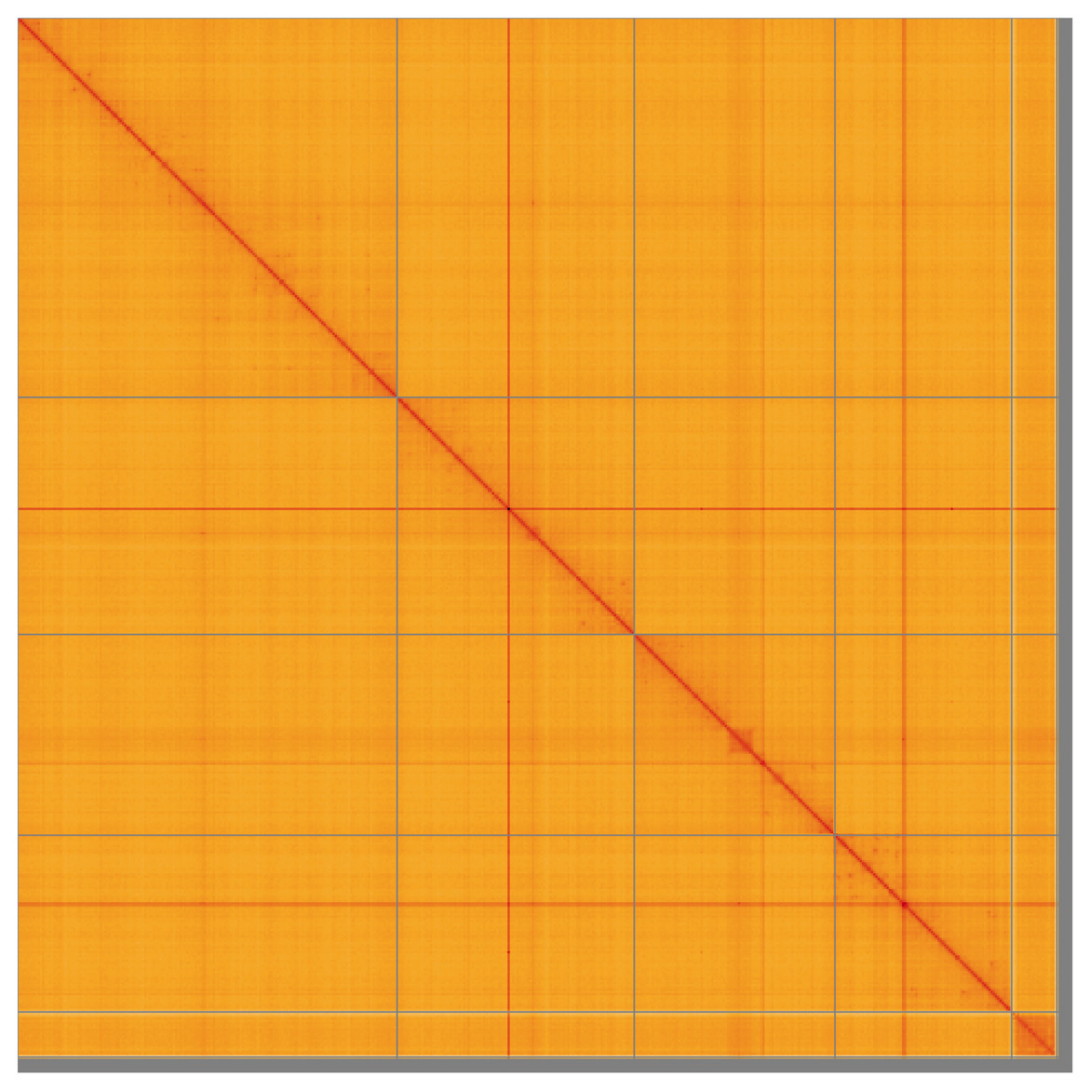
Genome assembly of *Toxonevra muliebris* idToxMuli2.1: Hi-C contact map of the idToxMuli2.1 assembly, visualised using HiGlass. Chromosomes are shown in order of size from left to right and top to bottom. An interactive version of this figure may be viewed at https://genome-note-higlass.tol.sanger.ac.uk/l/?d=ZEtLzfvEQ3GP4R-fPP3few.

Most of the assembly sequence (99.07%) was assigned to 6 chromosomal-level scaffolds, representing 4 autosomes and the X and Y sex chromosome. These chromosome-level scaffolds, confirmed by the Hi-C data, are named in order of size ( Figure 5; Table 3). During manual curation, chromosomes X and Y were assigned based on read coverage statistics. While not fully phased, the assembly deposited is of one haplotype. Contigs corresponding to the second haplotype have also been deposited.

**Table 3.** Chromosomal pseudomolecules in the genome assembly of *Toxonevra muliebris*, idToxMuli2.

INSDC accession	Name	Length (Mb)	GC%
OY829326.1	1	175.3	32.5
OY829327.1	2	109.56	32.5
OY829328.1	3	92.8	33.0
OY829329.1	4	81.68	33.0
OY829330.1	X	20.77	34.0
OY829331.1	Y	0.72	34.0
OY829332.1	MT	0.02	22.0

The mitochondrial genome was also assembled and can be found as a contig within the multifasta file of the genome submission, and as a separate fasta file with accession OY829332.1.

The final primary assembly has a Quality Value (QV) of 61.3. The *k*-mer completeness calculated in Merqury.FK is 84.35% for the primary assembly, 81.13% for the alternate haplotype, and 99.57% for the combined assemblies. BUSCO (v5.4.3) analysis using the diptera_odb10 reference set ( *n* = 3,285) indicated a completeness score of 98.7% (single = 97.7%, duplicated = 1.0%). The assembly achieves the EBP reference standard of 6.C.61. Other quality metrics are given in Table 2. 

## Genome annotation report

The *Toxonevra muliebris* genome assembly (GCA_963691655.1) was annotated at the European Bioinformatics Institute (EBI) on Ensembl Rapid Release. The resulting annotation includes 22,154 transcribed mRNAs from 21,433 protein-coding genes ( Table 2; https://rapid.ensembl.org/Toxonevra_muliebris_GCA_963691655.1/Info/Index). The average transcript length is 4,785.43, with 3.70 exons per transcript.

## Methods

### Sample acquisition and DNA barcoding

A male adult *Toxonevra muliebris* (specimen ID NHMUK014440495, ToLID idToxMuli2) was collected from William Parnell Park, Fulham, England, United Kingdom (latitude 51.47, longitude –0.18) on 2021-07-05 by hand picking. The specimen was collected and identified by Maxwell Barclay (Natural History Museum) and preserved by dry freezing at –80 °C.

The specimen used for Hi-C sequencing (specimen ID NHMUK010635000, ToLID idToxMuli1) was an adult specimen collected from England, Kent, Tonbridge, United Kingdom (latitude 51.19, longitude 0.29) on 2020-07-06. The specimen was collected and identified by Gavin Broad (Natural History Museum) and preserved on dry ice.

The initial identification was verified by an additional DNA barcoding process according to the framework developed by Twyford *et al*. (2024). A small sample was dissected from the specimens and stored in ethanol, while the remaining parts were shipped on dry ice to the Wellcome Sanger Institute (WSI). The tissue was lysed, the COI marker region was amplified by PCR, and amplicons were sequenced and compared to the BOLD database, confirming the species identification ( Crowley *et al.*, 2023). Following whole genome sequence generation, the relevant DNA barcode region was also used alongside the initial barcoding data for sample tracking at the WSI ( Twyford *et al.*, 2024). The standard operating procedures for Darwin Tree of Life barcoding have been deposited on protocols.io ( Beasley *et al.*, 2023).

### Nucleic acid extraction

The workflow for high molecular weight (HMW) DNA extraction at the Wellcome Sanger Institute (WSI) Tree of Life Core Laboratory includes a sequence of procedures: sample preparation and homogenisation, DNA extraction, fragmentation and purification. Detailed protocols are available on protocols.io ( Denton *et al.*, 2023b). The idToxMuli2 sample was prepared for DNA extraction by weighing and dissecting it on dry ice ( Jay *et al.*, 2023). Tissue from the abdomen was homogenised using a PowerMasher II tissue disruptor ( Denton *et al.*, 2023a). HMW DNA was extracted using the Automated MagAttract v2 protocol ( Oatley *et al.*, 2023a). For ULI PacBio sequencing, DNA was fragmented using the Covaris g-TUBE method ( Oatley *et al.*, 2023c). Sheared DNA was purified by solid-phase reversible immobilisation, using AMPure PB beads to eliminate shorter fragments and concentrate the DNA ( Oatley *et al.*, 2023b). The concentration of the sheared and purified DNA was assessed using a Nanodrop spectrophotometer and Qubit Fluorometer using the Qubit dsDNA High Sensitivity Assay kit. Fragment size distribution was evaluated by running the sample on the FemtoPulse system.

### Hi-C preparation

Tissue from the head of the dToxMuli1sample was processed at the WSI Scientific Operations core, using the Arima-HiC v2 kit. Tissue (stored at –80 °C) was fixed, and the DNA crosslinked using a TC buffer with 22% formaldehyde. After crosslinking, the tissue was homogenised using the Diagnocine Power Masher-II and BioMasher-II tubes and pestles. Following the kit manufacturer's instructions, crosslinked DNA was digested using a restriction enzyme master mix. The 5’-overhangs were then filled in and labelled with biotinylated nucleotides and proximally ligated. An overnight incubation was carried out for enzymes to digest remaining proteins and for crosslinks to reverse. A clean up was performed with SPRIselect beads prior to library preparation.

### Library preparation and sequencing

Library preparation and sequencing were performed at the WSI Scientific Operations Core.

***DNA (PacBio ULI)***

Ultra-low input sequencing (ULI) requires Covaris g-TUBE shearing to approximately 10 kb prior to library prep. ULI libraries were prepared using PacBio SMRTbell® Express Template Prep Kit 2.0 and PacBio SMRTbell® gDNA Sample Amplification Kit. To begin, samples were normalised to 20 ng of DNA. Initial removal of single-strand overhangs, DNA damage repair, and end repair/A-tailing were performed per manufacturer’s instructions. From the SMRTbell® gDNA Sample Amplification Kit, amplification adapters were then ligated. A 0.85X pre-PCR clean-up was performed with Promega ProNex beads and the sample was then divided into two for a dual PCR. PCR reactions A and B each followed the PCR programs as described in the manufacturer’s protocol. A 0.85X post-PCR clean-up was performed with ProNex beads for PCR reactions A and B and DNA concentration was quantified using the Qubit Fluorometer v2.0 (Thermo Fisher Scientific) and Qubit HS Assay Kit and fragment size analysis was carried out using the Agilent Femto Pulse Automated Pulsed Field CE Instrument (Agilent Technologies) and gDNA 55kb BAC analysis kit. PCR reactions A and B were then pooled, ensuring the total mass was ≥500 ng in 47.4 μl. The pooled sample then repeated the process for DNA damage repair, end repair/A-tailing and additional hairpin adapter ligation. A 1X clean-up was performed with ProNex beads and DNA concentration was quantified using the Qubit and fragment size analysis was carried out using the Agilent Femto Pulse Automated Pulsed Field CE Instrument (Agilent Technologies). Size selection was performed using Sage Sciences' PippinHT system with target fragment size determined by analysis from the Femto Pulse, usually a value between 4000 and 9000bp. Size selected libraries then had a final 1.0X clean up with ProNex beads and normalised to 2 nM before proceeding to sequencing.

Prepared libraries were normalised to 2 nM and 15 μL used for making complexes. For libraries below 2nM all 10uL was used for making complexes. Primers were annealed and polymerases were hybridised to create circularised complexes according to manufacturer’s instructions. The complexes were purified with the 1.2X clean up with SMRTbell beads. The purified complexes were then diluted to the Revio loading concentration, between 200 -300pM, and spiked with a Revio sequencing internal control. Samples were sequenced using the Revio system on Revio 25M SMRT cells (Pacific Biosciences, California, USA). The SMRT link software, a PacBio web-based end-to-end workflow manager, was used to set-up and monitor the run, as well as perform primary and secondary analysis of the data upon completion.

***Hi-C***

For Hi-C library preparation, DNA was fragmented to a size of 400 to 600 bp using a Covaris E220 sonicator. The DNA was then enriched, barcoded, and amplified using the NEBNext Ultra II DNA Library Prep Kit following manufacturers’ instructions. The Hi-C sequencing was performed using paired-end sequencing with a read length of 150 bp on an Illumina NovaSeq 6000 instrument.

### Genome assembly, curation and evaluation

***Assembly***

The HiFi reads were first assembled using Hifiasm ( Cheng *et al.*, 2021) with the --primary option. Haplotypic duplications were identified and removed using purge_dups ( Guan *et al.*, 2020). The Hi-C reads were mapped to the primary contigs using bwa-mem2 ( Vasimuddin *et al.*, 2019). The contigs were further scaffolded using the provided Hi-C data ( Rao *et al.*, 2014) in YaHS ( Zhou *et al.*, 2023) using the --break option for handling potential misassemblies. The scaffolded assemblies were evaluated using Gfastats ( Formenti *et al.*, 2022), BUSCO ( Manni *et al.*, 2021) and MERQURY.FK ( Rhie *et al.*, 2020).

The mitochondrial genome was assembled using MitoHiFi ( Uliano-Silva *et al.*, 2023), which runs MitoFinder ( Allio *et al.*, 2020) and uses these annotations to select the final mitochondrial contig and to ensure the general quality of the sequence.

***Assembly curation***

The assembly was decontaminated using the Assembly Screen for Cobionts and Contaminants (ASCC) pipeline (article in preparation). Flat files and maps used in curation were generated in TreeVal ( Pointon *et al.*, 2023). Manual curation was primarily conducted using PretextView ( Harry, 2022), with additional insights provided by JBrowse2 ( Diesh *et al.*, 2023) and HiGlass ( Kerpedjiev *et al.*, 2018). Scaffolds were visually inspected and corrected as described by Howe *et al*. (2021). Any identified contamination, missed joins, and mis-joins were corrected, and duplicate sequences were tagged and removed. The curation process is documented at https://gitlab.com/wtsi-grit/rapid-curation (article in preparation).

***Assembly quality assessment***

The Merqury.FK tool ( Rhie *et al.*, 2020) was used to evaluate *k*-mer completeness and assembly quality for the primary and alternate haplotypes using the *k*-mer databases ( *k* = 31) that were computed prior to genome assembly. The analysis outputs included assembly QV scores and completeness statistics.

A Hi-C contact map was produced for the final version of the assembly. The Hi-C reads were aligned using bwa-mem2 ( Vasimuddin *et al.*, 2019) and the alignment files were combined using SAMtools ( Danecek *et al.*, 2021). The Hi-C alignments were converted into a contact map using BEDTools ( Quinlan & Hall, 2010) and the Cooler tool suite ( Abdennur & Mirny, 2020). The contact map is visualised in HiGlass ( Kerpedjiev *et al.*, 2018).

The blobtoolkit pipeline is a Nextflow ( Di Tommaso *et al.*, 2017) port of the previous Snakemake Blobtoolkit pipeline ( Challis *et al.*, 2020). It aligns the PacBio reads in SAMtools and minimap2 ( Li, 2018) and generates coverage tracks for regions of fixed size. In parallel, it queries the GoaT database ( Challis *et al.*, 2023) to identify all matching BUSCO lineages to run BUSCO ( Manni *et al.*, 2021). For the three domain-level BUSCO lineages, the pipeline aligns the BUSCO genes to the UniProt Reference Proteomes database ( Bateman *et al.*, 2023) with DIAMOND blastp ( Buchfink *et al.*, 2021). The genome is also divided into chunks according to the density of the BUSCO genes from the closest taxonomic lineage, and each chunk is aligned to the UniProt Reference Proteomes database using DIAMOND blastx. Genome sequences without a hit are chunked using seqtk and aligned to the NT database with blastn ( Altschul *et al.*, 1990). The blobtools suite combines all these outputs into a blobdir for visualisation.

The blobtoolkit pipeline was developed using nf-core tooling ( Ewels *et al.*, 2020) and MultiQC ( Ewels *et al.*, 2016), relying on the Conda package manager, the Bioconda initiative ( Grüning *et al.*, 2018), the Biocontainers infrastructure ( da Veiga Leprevost *et al.*, 2017), as well as the Docker ( Merkel, 2014) and Singularity ( Kurtzer *et al.*, 2017) containerisation solutions.

Table 4 contains a list of relevant software tool versions and sources.

**Table 4.** Software tools: versions and sources.

Software tool	Version	Source
BEDTools	2.30.0	https://github.com/arq5x/bedtools2
BLAST	2.14.0	ftp://ftp.ncbi.nlm.nih.gov/blast/executables/blast+/
BlobToolKit	4.3.7	https://github.com/blobtoolkit/blobtoolkit
BUSCO	5.4.3 and 5.5.0	https://gitlab.com/ezlab/busco
bwa-mem2	2.2.1	https://github.com/bwa-mem2/bwa-mem2
Cooler	0.8.11	https://github.com/open2c/cooler
DIAMOND	2.1.8	https://github.com/bbuchfink/diamond
fasta_windows	0.2.4	https://github.com/tolkit/fasta_windows
FastK	427104ea91c78c3b8b8b49f1a7d6bbeaa869ba1c	https://github.com/thegenemyers/FASTK
Gfastats	1.3.6	https://github.com/vgl-hub/gfastats
GoaT CLI	0.2.5	https://github.com/genomehubs/goat-cli
Hifiasm	0.19.8-r587	https://github.com/chhylp123/hifiasm
HiGlass	44086069ee7d4d3f6f3f0012569789ec138f42b84a a44357826c0b6753eb28de	https://github.com/higlass/higlass
Merqury.FK	d00d98157618f4e8d1a9190026b19b471055b22e	https://github.com/thegenemyers/MERQURY.FK
MitoHiFi	3	https://github.com/marcelauliano/MitoHiFi
MultiQC	1.14, 1.17, and 1.18	https://github.com/MultiQC/MultiQC
NCBI Datasets	15.12.0	https://github.com/ncbi/datasets
Nextflow	23.04.0-5857	https://github.com/nextflow-io/nextflow
PretextView	0.2.5	https://github.com/sanger-tol/PretextView
purge_dups	1.2.5	https://github.com/dfguan/purge_dups
samtools	1.16.1, 1.17, and 1.18	https://github.com/samtools/samtools
sanger-tol/ascc	-	https://github.com/sanger-tol/ascc
Seqtk	1.3	https://github.com/lh3/seqtk
Singularity	3.9.0	https://github.com/sylabs/singularity
TreeVal	1.0.0	https://github.com/sanger-tol/treeval
YaHS	1.2a.2	https://github.com/c-zhou/yahs

### Genome annotation

The BRAKER2 pipeline ( Brůna *et al.*, 2021) was used in the default protein mode to generate annotation for the *Toxonevra muliebris* assembly (GCA_963691655.1) in Ensembl Rapid Release at the EBI.

### Wellcome Sanger Institute – Legal and Governance

The materials that have contributed to this genome note have been supplied by a Darwin Tree of Life Partner. The submission of materials by a Darwin Tree of Life Partner is subject to the **‘Darwin Tree of Life Project Sampling Code of Practice’**, which can be found in full on the Darwin Tree of Life website here. By agreeing with and signing up to the Sampling Code of Practice, the Darwin Tree of Life Partner agrees they will meet the legal and ethical requirements and standards set out within this document in respect of all samples acquired for, and supplied to, the Darwin Tree of Life Project.

Further, the Wellcome Sanger Institute employs a process whereby due diligence is carried out proportionate to the nature of the materials themselves, and the circumstances under which they have been/are to be collected and provided for use. The purpose of this is to address and mitigate any potential legal and/or ethical implications of receipt and use of the materials as part of the research project, and to ensure that in doing so we align with best practice wherever possible. The overarching areas of consideration are:

• Ethical review of provenance and sourcing of the material

• Legality of collection, transfer and use (national and international)

Each transfer of samples is further undertaken according to a Research Collaboration Agreement or Material Transfer Agreement entered into by the Darwin Tree of Life Partner, Genome Research Limited (operating as the Wellcome Sanger Institute), and in some circumstances other Darwin Tree of Life collaborators.

## Data Availability

European Nucleotide Archive: Toxoneura muliebris (looped flutter fly). Accession number PRJEB68022; https://identifiers.org/ena.embl/PRJEB68022. The genome sequence is released openly for reuse. The *Toxonevra muliebris* genome sequencing initiative is part of the Darwin Tree of Life (DToL) project. All raw sequence data and the assembly have been deposited in INSDC databases. Raw data and assembly accession identifiers are reported in Table 1 and Table 2.

## Author information

Members of the Natural History Museum Genome Acquisition Lab are listed here: https://doi.org/10.5281/zenodo.12159242.

Members of the Darwin Tree of Life Barcoding collective are listed here: https://doi.org/10.5281/zenodo.12158331.

Members of the Wellcome Sanger Institute Tree of Life Management, Samples and Laboratory team are listed here: https://doi.org/10.5281/zenodo.12162482.

Members of Wellcome Sanger Institute Scientific Operations: Sequencing Operations are listed here: https://doi.org/10.5281/zenodo.12165051.

Members of the Wellcome Sanger Institute Tree of Life Core Informatics team are listed here: https://doi.org/10.5281/zenodo.12160324.

Members of the Tree of Life Core Informatics collective are listed here: https://doi.org/10.5281/zenodo.12205391.

Members of the Darwin Tree of Life Consortium are listed here: https://doi.org/10.5281/zenodo.4783558.

## References

[ref-1] Abdennur N Mirny LA : Cooler: scalable storage for Hi-C data and other genomically labeled arrays. *Bioinformatics.* 2020;36(1):311–316. 10.1093/bioinformatics/btz540 31290943 PMC8205516

[ref-2] Allio R Schomaker-Bastos A Romiguier J : MitoFinder: efficient automated large-scale extraction of mitogenomic data in target enrichment phylogenomics. *Mol Ecol Resour.* 2020;20(4):892–905. 10.1111/1755-0998.13160 32243090 PMC7497042

[ref-3] Altschul SF Gish W Miller W : Basic local alignment search tool. *J Mol Biol.* 1990;215(3):403–410. 10.1016/S0022-2836(05)80360-2 2231712

[ref-4] Bateman A Martin MJ Orchard S : UniProt: the Universal Protein Knowledgebase in 2023. *Nucleic Acids Res.* 2023;51(D1):D523–D531. 10.1093/nar/gkac1052 36408920 PMC9825514

[ref-5] Beasley J Uhl R Forrest LL : DNA barcoding SOPs for the Darwin Tree of Life project. *protocols.io.* 2023; [Accessed 25 June 2024]. 10.17504/protocols.io.261ged91jv47/v1

[ref-6] Brůna T Hoff KJ Lomsadze A : BRAKER2: automatic eukaryotic genome annotation with GeneMark-EP+ and AUGUSTUS supported by a protein database. *NAR Genom Bioinform.* 2021;3(1): lqaa108. 10.1093/nargab/lqaa108 33575650 PMC7787252

[ref-7] Buchfink B Reuter K Drost HG : Sensitive protein alignments at Tree-of-Life scale using DIAMOND. *Nat Methods.* 2021;18(4):366–368. 10.1038/s41592-021-01101-x 33828273 PMC8026399

[ref-8] Cannings RA Gibson JF : *Toxonevra muliebris* (Harris) (Diptera: Pallopteridae): a European fly new to North America. *J Entomol Soc B C.* 2019;116:64–68. Reference Source

[ref-9] Challis R Kumar S Sotero-Caio C : Genomes on a Tree (GoaT): a versatile, scalable search engine for genomic and sequencing project metadata across the eukaryotic Tree of Life [version 1; peer review: 2 approved]. *Wellcome Open Res.* 2023;8:24. 10.12688/wellcomeopenres.18658.1 36864925 PMC9971660

[ref-10] Challis R Richards E Rajan J : BlobToolKit – interactive quality assessment of genome assemblies. *G3 (Bethesda).* 2020;10(4):1361–1374. 10.1534/g3.119.400908 32071071 PMC7144090

[ref-11] Chandler PJ : Attraction of *Palloptera usta* Meigen (Diptera: Pallopteridae) to recently cut conifer wood and other notes on Pallopteridae. *Brit J Entomol Nat Hist.* 1991;4(2):85–86.

[ref-12] Chandler PJ : (Ed.) An update of the 1998 checklist of Diptera of the British Isles.Dipterists Forum,2024; [updated 18 April 2024]. Reference Source

[ref-13] Cheng H Concepcion GT Feng X : Haplotype-resolved *de novo* assembly using phased assembly graphs with hifiasm. *Nat Methods.* 2021;18(2):170–175. 10.1038/s41592-020-01056-5 33526886 PMC7961889

[ref-14] Collin JE : The British Species of the Genus *Palloptera* Fallen (Diptera). *Entomol Record.* 1951;63:1–6.

[ref-15] Crowley L Allen H Barnes I : A sampling strategy for genome sequencing the British terrestrial arthropod fauna [version 1; peer review: 2 approved]. *Wellcome Open Res.* 2023;8:123. 10.12688/wellcomeopenres.18925.1 37408610 PMC10318377

[ref-16] da Veiga Leprevost F Grüning BA Alves Aflitos S : BioContainers: an open-source and community-driven framework for software standardization. *Bioinformatics.* 2017;33(16):2580–2582. 10.1093/bioinformatics/btx192 28379341 PMC5870671

[ref-17] Danecek P Bonfield JK Liddle J : Twelve years of SAMtools and BCFtools. *GigaScience.* 2021;10(2): giab008. 10.1093/gigascience/giab008 33590861 PMC7931819

[ref-18] Denton A Oatley G Cornwell C : Sanger Tree of Life sample homogenisation: PowerMash. *protocols.io.* 2023a. 10.17504/protocols.io.5qpvo3r19v4o/v1

[ref-19] Denton A Yatsenko H Jay J : Sanger Tree of Life wet laboratory protocol collection V.1. *protocols.io.* 2023b. 10.17504/protocols.io.8epv5xxy6g1b/v1

[ref-20] Di Tommaso P Chatzou M Floden EW : Nextflow enables reproducible computational workflows. *Nat Biotechnol.* 2017;35(4):316–319. 10.1038/nbt.3820 28398311

[ref-21] Diesh C Stevens GJ Xie P : JBrowse 2: a modular genome browser with views of synteny and structural variation. *Genome Biol.* 2023;24(1): 74. 10.1186/s13059-023-02914-z 37069644 PMC10108523

[ref-23] Ewels P Magnusson M Lundin S : MultiQC: summarize analysis results for multiple tools and samples in a single report. *Bioinformatics.* 2016;32(19):3047–3048. 10.1093/bioinformatics/btw354 27312411 PMC5039924

[ref-22] Ewels PA Peltzer A Fillinger S : The nf-core framework for community-curated bioinformatics pipelines. *Nat Biotechnol.* 2020;38(3):276–278. 10.1038/s41587-020-0439-x 32055031

[ref-24] Formenti G Abueg L Brajuka A : Gfastats: conversion, evaluation and manipulation of genome sequences using assembly graphs. *Bioinformatics.* 2022;38(17):4214–4216. 10.1093/bioinformatics/btac460 35799367 PMC9438950

[ref-25] Griffiths GCD : The Phylogenetic Classification of Diptera Cyclorrhapha, with Special Reference to the Structure of the Male Postabdomen.Dr. W. Junk, N. V., The Hague,1972;8. 10.1007/978-94-015-7243-9

[ref-26] Grüning B Dale R Sjödin A : Bioconda: sustainable and comprehensive software distribution for the life sciences. *Nat Methods.* 2018;15(7):475–476. 10.1038/s41592-018-0046-7 29967506 PMC11070151

[ref-27] Guan D McCarthy SA Wood J : Identifying and removing haplotypic duplication in primary genome assemblies. *Bioinformatics.* 2020;36(9):2896–2898. 10.1093/bioinformatics/btaa025 31971576 PMC7203741

[ref-28] Han HY Ro KE : Molecular phylogeny of the superfamily Tephritoidea (Insecta: Diptera) reanalysed based on expanded taxon sampling and sequence data. *J Zool Syst Evol Res.* 2016;54(4):276–288. 10.1111/jzs.12139

[ref-29] Harry E : PretextView (Paired REad TEXTure Viewer): a desktop application for viewing pretext contact maps.2022. Reference Source

[ref-30] Howe K Chow W Collins J : Significantly improving the quality of genome assemblies through curation. *GigaScience.* 2021;10(1): giaa153. 10.1093/gigascience/giaa153 33420778 PMC7794651

[ref-31] Jay J Yatsenko H Narváez-Gómez JP : Sanger Tree of Life sample preparation: triage and dissection. *protocols.io.* 2023. 10.17504/protocols.io.x54v9prmqg3e/v1

[ref-32] Jones R : House Guests, House Pests: a natural history of animals in the home.Bloomsbury Publishing,2015. Reference Source

[ref-33] Kerpedjiev P Abdennur N Lekschas F : HiGlass: web-based visual exploration and analysis of genome interaction maps. *Genome Biol.* 2018;19(1): 125. 10.1186/s13059-018-1486-1 30143029 PMC6109259

[ref-34] Kurtzer GM Sochat V Bauer MW : Singularity: scientific containers for mobility of compute. *PLoS One.* 2017;12(5): e0177459. 10.1371/journal.pone.0177459 28494014 PMC5426675

[ref-35] Li H : Minimap2: pairwise alignment for nucleotide sequences. *Bioinformatics.* 2018;34(18):3094–3100. 10.1093/bioinformatics/bty191 29750242 PMC6137996

[ref-36] Manni M Berkeley MR Seppey M : BUSCO update: novel and streamlined workflows along with broader and deeper phylogenetic coverage for scoring of eukaryotic, prokaryotic, and viral genomes. *Mol Biol Evol.* 2021;38(10):4647–4654. 10.1093/molbev/msab199 34320186 PMC8476166

[ref-37] Marshall SA : Flies: the natural history and diversity of Diptera.Firefly Books, Richmond Hill, Ontario, Canada,2012. Reference Source

[ref-38] McAlpine F : *Morgea freidbergi* new sp., a living sister species of the fossil species *M. mcalpinei* and a key to World genera of Pallopteridae (Diptera). *Can Entomol.* 1981;113:81–91.

[ref-39] Merkel D : Docker: lightweight Linux containers for consistent development and deployment. *Linux J.* 2014;2014(239): 2, [Accessed 2 April 2024]. Reference Source

[ref-40] NBN Atlas Partnership: *Toxonevra muliebris* (Harris, 1780) map on the NBN Atlas. 2024. Reference Source

[ref-41] Oatley G Denton A Howard C : Sanger Tree of Life HMW DNA extraction: automated MagAttract v.2. *protocols.io.* 2023a. 10.17504/protocols.io.kxygx3y4dg8j/v1

[ref-42] Oatley G Sampaio F Howard C : Sanger Tree of Life fragmented DNA clean up: automated SPRI. *protocols.io.* 2023b. 10.17504/protocols.io.q26g7p1wkgwz/v1

[ref-43] Oatley G Sampaio F Kitchin L : Sanger Tree of Life HMW DNA fragmentation: Covaris g-TUBE for ULI PacBio. *protocols.io.* 2023c; [Accessed 13 June 2024]. 10.17504/protocols.io.q26g7pm81gwz/v1

[ref-44] Papp L : Description of a new genus and a new family Circumphallidae Fam. Nov., of the acalyptrate flies (Diptera). *Acta Zool Acad Sci Hung.* 2011;57(4):315–341. Reference Source

[ref-45] Parmenter L : More Flies of the Cripplegate Bombed Site, City of London. *London Nat, J London Nat Hist Soc.* 1968;47:81–86.

[ref-46] Pointon DL Eagles W Sims Y : sanger-tol/treeval v1.0.0 – Ancient Atlantis. 2023. 10.5281/zenodo.10047654

[ref-47] Quinlan AR Hall IM : BEDTools: a flexible suite of utilities for comparing genomic features. *Bioinformatics.* 2010;26(6):841–842. 10.1093/bioinformatics/btq033 20110278 PMC2832824

[ref-48] Rao SSP Huntley MH Durand NC : A 3D map of the human genome at kilobase resolution reveals principles of chromatin looping. *Cell.* 2014;159(7):1665–1680. 10.1016/j.cell.2014.11.021 25497547 PMC5635824

[ref-49] Rhie A McCarthy SA Fedrigo O : Towards complete and error-free genome assemblies of all vertebrate species. *Nature.* 2021;592(7856):737–746. 10.1038/s41586-021-03451-0 33911273 PMC8081667

[ref-50] Rhie A Walenz BP Koren S : Merqury: reference-free quality, completeness, and phasing assessment for genome assemblies. *Genome Biol.* 2020;21(1): 245. 10.1186/s13059-020-02134-9 32928274 PMC7488777

[ref-51] Rotheray GE : Development sites, feeding modes and early stages of seven European *Palloptera* species (Diptera, Pallopteridae). *Zootaxa.* 2014;3900(1):50–76. 10.11646/zootaxa.3900.1.3 25543723

[ref-52] Speight MCD : *Cheilosia argentifrons* (Diptera: Syrphidae) new to Ireland: *Donacia cinerea* (Coleoptera: Chrysomelidae) and *Palloptera muliebris* (Diptera: Pallopteridae), presence confirmed in Ireland. *Irish Nat J.* 1986;22:159–160.

[ref-53] Stubbs AE Clements DK : Revised draft key to British Pallopteridae. *Picture-Winged Flies Recording Scheme Newsletter*,1999.

[ref-54] Twyford AD Beasley J Barnes I : A DNA barcoding framework for taxonomic verification in the Darwin Tree of Life project [version 1; peer review: 2 approved]. *Wellcome Open Res.* 2024;9:339. 10.12688/wellcomeopenres.21143.1 39386966 PMC11462125

[ref-55] Uliano-Silva M Ferreira JGRN Krasheninnikova K : MitoHiFi: a python pipeline for mitochondrial genome assembly from PacBio high fidelity reads. *BMC Bioinformatics.* 2023;24(1): 288. 10.1186/s12859-023-05385-y 37464285 PMC10354987

[ref-56] Vasimuddin M Misra S Li H : Efficient architecture-aware acceleration of BWA-MEM for multicore systems.In: *2019 IEEE International Parallel and Distributed Processing Symposium (IPDPS).*IEEE,2019;314–324. 10.1109/IPDPS.2019.00041

[ref-57] Wallace PF O’Connor JP : *Palloptera muliebris* (Harris) (Dipt., Pallopteridae) discovered in Dublin City. *Entomol Mon Mag.* 1997;133:114.

[ref-58] Weddle RB : *Palloptera muliebris* (Diptera: Pallopteridae): a rare Scottish occurrence. *Glasgow Nat.* 2019;27:90–91. 10.37208/tgn27126

[ref-59] Zhou C McCarthy SA Durbin R : YaHS: yet another Hi-C scaffolding tool. *Bioinformatics.* 2023;39(1): btac808. 10.1093/bioinformatics/btac808 36525368 PMC9848053

