# Supplementary material for: The genome sequence of a pallopterid fly, Toxonevra muliebris (Harris, 1780)
Source: Wellcome Open Res. 2025 Feb 10;10:55. [Version 1] doi: 10.12688/wellcomeopenres.23670.1 (PMC11880760; doi:10.12688/wellcomeopenres.23670.1)
Supplement: Peer review history

# Reviewer report: Reviewer response for version 1

**DOI:** 10.21956/wellcomeopenres.26113.r118565

**Published:** 2025-03-04

**Authors:** Thomas Brown

**Affiliations:** 1 Leibniz Institute for Zoo and Wildlife Research (IZW) in the Forschungsverbund Berlin eV, Berlin, Berlin, Germany

Barclay et al present a high quality genome for the fly *Toxonevra muliebris*. The rational for why a genome is necessary for this species as well as the methods and results section are well written. There are a couple of small areas that I feel either need addressing, or would help the readers in interpreting the data for its future use.

 The sex in the listed BioSample for the PacBio data is stated as "NOT PROVIDED". It would be helpful to update this to male, as is stated in the manuscript.

 The BioSample for the Hi-C data is stated as female. This explains the lack of Hi-C signal in the Higlass image in Figure 5, but is a little misleading to say that the Y is fully scaffolded. I suspect a lot of unscaffolded sequences should belong to the Y chromosome, but have not been incorporated due to the missing data. This should be made clearer in the text.

 I would suggest including completeness statistics regarding the protein-coding annotation, such as running BUSCO in protein mode, or OMArk.

 It is not stated in the genome annotation methods which proteins were used to generate the gene models using braker2.

Are sufficient details of methods and materials provided to allow replication by others?

Partly

Is the rationale for creating the dataset(s) clearly described?

Yes

Are the datasets clearly presented in a useable and accessible format?

Yes

Are the protocols appropriate and is the work technically sound?

Yes

Reviewer Expertise:

Genome assembly & annotation. Bioinformatics. Workflows

I confirm that I have read this submission and believe that I have an appropriate level of expertise to confirm that it is of an acceptable scientific standard.

# Reviewer report: Reviewer response for version 1

**DOI:** 10.21956/wellcomeopenres.26113.r118561

**Published:** 2025-02-18

**Authors:** Ruiqi Li

**Affiliations:** 1 University of Colorado Boulder, Boulder, Colorado, USA

Barclay et al. presents a high quality genome of *Toxonevra muliebris. *I think the methodology is rigorous and the output is of high quality. I only have a few minor comments:

 1. In the introductions, it would be an addition if the authors could identify scientific questions that this genome can help us to answer. 

 2. In "Sample acquisition and DNA barcoding", it would be great if the authors could provide more details on how the specimens were identified (based on what morphological features).

Are sufficient details of methods and materials provided to allow replication by others?

Yes

Is the rationale for creating the dataset(s) clearly described?

Yes

Are the datasets clearly presented in a useable and accessible format?

Yes

Are the protocols appropriate and is the work technically sound?

Yes

Reviewer Expertise:

Genomics

I confirm that I have read this submission and believe that I have an appropriate level of expertise to confirm that it is of an acceptable scientific standard.
